# Supplementary material for: Assessing the Impact of Deep Neural Network-Based Image Denoising on Binary Signal Detection Tasks
Source: IEEE Trans Med Imaging. Author manuscript; Available in PMC 2021 Dec 15. (PMC8673589; doi:10.1109/TMI.2021.3076810)
Supplement: supp1-3076810 [file NIHMS1737262-supplement-supp1-3076810.pdf]

Supplementary Material

1 Propagation of covariance matrix through the linear denoising network

A covariance matrix propagation strategy was employed in Sec. III.C.1 in the main article to evaluate the RHO performance at the output of each layer of the linear denoising networks. The strategy avoids the need to empirically estimate the covariance matrix corresponding to the output of each layer of the linear denoising network. The strategy details are described below.

1.1 Linear convolutional layer

The vectorized tensor-valued data and the associated covariance matrices corresponding to each layer were computed by propagating the corresponding quantites from the previous layer. Let $\mathbf{k}_d \in \mathbb{R}^{c_{out} \times c_{in} \times h \times w}$ denote the convolution kernel of the dth convolutional layer. Here, d denotes the index of network depth, h and w denote the height and width of $\mathbf{k}_d$, c_{in} denotes the number of channels of the input tensor $\mathbf{g}_{d-1}$, and c_{out} denotes the number of channels in the output tensor $\mathbf{g}_d$. Let $\mathbf{g}_{d-1} \in \mathbb{R}^{c_{in} \times N \times N}$ denote the corresponding input tensor and $\mathbf{g}_d \in \mathbb{R}^{c_{out} \times N \times N}$ denote the corresponding output tensor, respectively, where $N \times N$ denotes the spatial dimension of $\mathbf{g}_{d-1}$. The output $\mathbf{g}_d$ is defined to have the same spatial dimensions (i.e., $N \times N$) as the input $\mathbf{g}_{d-1}$, accomplished by use of zero padding. The output of a convolutional layer can be expressed as:

$$\mathbf{g}_d = \mathbf{k}_d * \mathbf{g}_{d-1} + \mathbf{b}_d, \tag{S.1}$$

where $*$ denotes the multi-channel convolution operation employed by convolutional layers and $\mathbf{b}_d \in \mathbb{R}^{c_{out} \times N \times N}$ is the bias term of the dth convolutional layer. Let $vec(\cdot)$ represent a vectorization function that forms a column vector by stacking the columns of the flattening of an image tensor along the first dimension (see details in [1]). Equation (S.1) can be written as a matrix-vector product [1,2]:

$$vec(\mathbf{g}_d) = \mathbb{M}_d vec(\mathbf{g}_{d-1}) + vec(\mathbf{b}_d), \tag{S.2}$$

where $\mathbb{M}_d \in \mathbb{R}^{c_{out}N^2 \times c_{in}N^2}$ is the corresponding linear transformation matrix of the convolution kernel $\mathbf{k}_d$. The covariance matrix $\mathbf{K}_d \in \mathbb{R}^{c_{out}N^2 \times c_{out}N^2}$ of the output image tensor can be determined based on Eqn. (S.2):

$$\mathbf{K}_d = \mathbb{M}_d \mathbf{K}_{d-1} \mathbb{M}_d^{\dagger}. \tag{S.3}$$

Here $\mathbb{M}_d$ can be represented as a multi-block multi-level (doubly) Toeplitz matrix:

$$\mathbb{M}_d \equiv \mathbb{M}_d(\mathbf{k}_d) = \begin{bmatrix} \mathcal{M}_{1,1} & \mathcal{M}_{1,2} & \dots & \mathcal{M}_{1,c_{in}} \\ \mathcal{M}_{2,1} & \mathcal{M}_{2,2} & \dots & \mathcal{M}_{2,c_{in}} \\ \vdots & \vdots & \ddots & \vdots \\ \mathcal{M}_{c_{out},1} & \mathcal{M}_{c_{out},2} & \dots & \mathcal{M}_{c_{out},c_{in}} \end{bmatrix}, \tag{S.4}$$

where each block $\mathcal{M}_{i,j}$ in $\mathbb{M}$ is a $N^2 \times N^2$ doubly Toeplitz matrix that can be described as:

$$\mathcal{M}_{i,j} = \begin{bmatrix} \mathbf{M}_0^{i,j} & \dots & \mathbf{M}_{-h_1}^{i,j} & 0 & \dots & 0 \\ \vdots & \mathbf{M}_0^{i,j} & \ddots & \ddots & \ddots & \vdots \\ \mathbf{M}_{h_2}^{i,j} & \ddots & \ddots & \ddots & \ddots & 0 \\ 0 & \ddots & \ddots & \ddots & \ddots & \mathbf{M}_{-h_1}^{i,j} \\ \vdots & \ddots & \ddots & \ddots & \mathbf{M}_0^{i,j} & \vdots \\ 0 & \dots & 0 & \mathbf{M}_{h_2}^{i,j} & \dots & \mathbf{M}_0^{i,j} \end{bmatrix}. \tag{S.5}$$

Here, $i \in [1, c_{out}]$, $j \in [1, c_{in}]$, h_1 and h_2 are related to the size of padding in height subject to $h = h_1 + h_2 + 1$. Each block $\mathbf{M}_k^{i,j}$ in $\mathcal{M}_{i,j}$ is a banded Toeplitz matrix given by:

$$\mathbf{M}_k^{i,j} = \begin{bmatrix} m_{k,0}^{i,j} & \dots & m_{k,w_2}^{i,j} & 0 & \dots & 0 \\ \vdots & m_{k,0}^{i,j} & \ddots & \ddots & \ddots & \vdots \\ m_{k,-w_1}^{i,j} & \ddots & \ddots & \ddots & \ddots & 0 \\ 0 & \ddots & \ddots & \ddots & \ddots & m_{k,w_2}^{i,j} \\ \vdots & \ddots & \ddots & \ddots & m_{k,0}^{i,j} & \vdots \\ 0 & \dots & 0 & m_{k,-w_1}^{i,j} & \dots & m_{k,0}^{i,j} \end{bmatrix}, \tag{S.6}$$

where w_1 and w_2 subject to $w = w_1 + w_2 + 1$ that are determined by size of padding in width, and k ranges from $-h_1$ to h_2. The elements $m_{k,l}^{i,j}$ in $\mathbf{M}_k^{i,j}$ are the corresponding elements of the convolution kernel $\mathbf{k}_d$ and can be written as:

$$m_{k,l}^{i,j} = \mathbf{k}_{d_{i,j,(h_1+k+1),(w_1+l+1)}}, \tag{S.7}$$

where $\mathbf{k}_{d_{i,j,(h_1+k+1),(w_1+l+1)}}$ denotes the related element in $\mathbf{k}_d$, of which $l \in [-w_1, w_2]$.

1.2 Propagation of covariance matrix

Based on Eqn. (S.3) above and Eqn. (5) in the main article, the RHO detection performance at the output of each intermediate layer of the linear network can be computed according to the Algorithm 1 below.

Algorithm 1: RHO detection performance propagation along the linear denoising network

Input: An ensemble of noisy measurement $\mathbf{g}_0$, the covariance matrix $\mathbf{K}_0$, the linear denoising network with depth D, and the convolution kernels $\mathbf{k}_d$, in which d denotes the index of depth.

Output: RHO performance on the outputs of each intermediate layer d of the denoising network, denoted by AUC_d.

```
Calculate Δḡ_0;    // Difference between the mean of
    the ensemble of noisy measurements under the
    signal-present and signal-absent hypotheses.
for d=1:D do
    Calculate the linear transformation matrix M_d by use
      of k_d and based on Eqns. (S.4)-(S.7);
    K_d = M_d K_{d-1} M_d^†;    // Calculate the covariance
      matrix through an linear transformation
    vec(g_d) = M_d vec(g_{d-1}) + vec(b_d);
    vec(Δḡ_d) = M_d vec(Δḡ_{d-1});
    Calculate the RHO test statistic t_RHO(g_d) with a
      pre-defined λ by use of Eqn. (S.3) and Eqn. (5) in the
      main article;
    Generate the ROC curve and calculate the
      corresponding AUC value (AUC_d)
```

2 Objective evaluation of denoising networks trained by use of low-noise target images

The CNN-based nonlinear denoising networks, described in Sec. III.B.2 in the main article, were retrained by use of low-noise images, instead of noise-free ones, as the target images. The low-noise images were created by adding object-dependent Poisson noise into the noise-free images described in Sec. III.C.2 in the main article. The same training/validation/testing scheme

described in Sec. III.B.4 was employed for training. The CNN-based denoising networks with the depth of {3, 5, 7, 9, 11, 13} respectively were employed in this study. The impact of depth of the trained denoising network on the NO performance was shown in Fig. S.1. It was observed that the NO performance on the output images yielded the same trends as were observed for the case where noise-free images were employed to train the denoising networks. As such, these results are consistent with those shown in Fig. 6 in the main article.

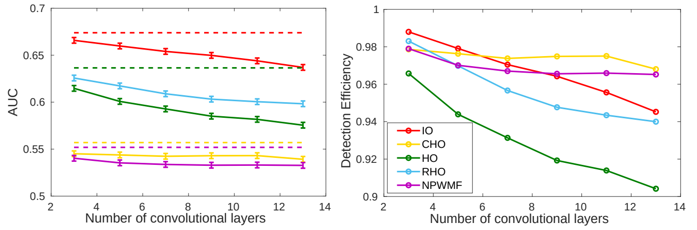

Figure S.1: The relationship between NO performance and the depth of the trained CNN-based non-linear denoising networks was quantified. The CNN-based denoising networks with the depth of {3, 5, 7, 9, 11, 13} respectively were employed in this study. The observer performance is quantified by AUC and signal detection efficiency. The two figures share the same legend and the dashed lines in the left figures represent the detection performance of NOs on the noisy images.

3 Detection efficiency vs. noise level

To determine the effects of measurement noise on signal detectability, the performance of the HO on the original noisy images and noise-free target images was compared. The settings were the same as described in Sec. III.C.2 in the main article. It was observed that the HO performed perfectly (AUC=1) on the noise-free images, while the AUC values dropped significantly (AUC=0.62 $\pm$ 0.003) when acting on the noisy images. This demonstrates that, in the cases considered, measurement noise was the dominant stochastic factor that limited signal detectability.

The impact of noise level on RHO detection efficiency is shown in Fig. S.2. The imaging system, background, and signal employed here were the same as described in Sec. III.C.2 in the main article. The mixed Poisson-Gaussian noise was employed with different choices of the standard deviation of the Gaussian noise: {15, 35, 55, 75}. The CNN-based denoising networks with the depth of {3, 5, 7, 9, 11, 13} were employed in this study. It was observed that, for each noise level, the detection efficiency was reduced as the denoising network depth increased. Additionally, the detection efficiency reduced more rapidly as a function of network depth for smaller noise levels as compared to larger ones. When the effects from measurement noise on signal detectability become larger (the effects from background variability become smaller), the degradation of signal detection performance due to the denoising operation become smaller.

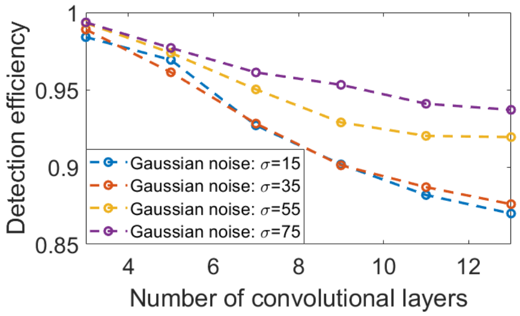

Figure S.2: The relationship between signal size and RHO detection efficiency was quantified. Here, the CNN-based denoising method was employed with an MSE loss and the network depth was varied: $D = \{3, 5, 7, 9, 11, 13\}$. Detection efficiency reduced more rapidly as a function of network depth when the signal size was reduced.

4 Objective evaluation of nonlinear denoising networks using simulated MR images

4.1 Simulated magnetic resonance (MR) images and binary signal detection task

A stylized MRI system that acquires fully-sampled k-space data was employed to simulate magnetic resonance (MR) images. The imaging process was described as:

$$\mathbf{g} = \mathbf{H}\mathbf{f} + \mathbf{n}, \tag{S.8a}$$

$$\mathbf{f}_{recon} = |\mathbf{H}^{-1}\mathbf{g}|, \tag{S.8b}$$

where $\mathbf{g}$ denotes the k-space noisy measurements, $\mathbf{f}_{recon}$ denotes the reconstructed noisy magnitude images, and $\mathbf{H}$ denotes a 2D discrete Fourier transform (DFT) matrix. The measurement noise $\mathbf{n}$ followed a complex-valued Gaussian distribution considering the physical properties of MRI.

The noisy measurement $\mathbf{g}$ was simulated by computing the 2D DFT of the object $\mathbf{f}$ and adding i.i.d. zero mean Gaussian noise to both the real and imaginary components of the k-space data. The reconstructed noisy images $\mathbf{f}_{recon}$ were formed by acting a 2D inverse DFT (IDFT) to each measured image data $\mathbf{g}$ and taking the absolute value.

The signal object $\mathbf{f_s}$ was a deterministic Gaussian image and the mth element $\mathbf{f_s}^m$ of the signal object $\mathbf{f_s}$ can be computed as:

$$\mathbf{f_s}^m = A_s \exp\left[-\frac{(\mathbf{r}_m - \mathbf{r}_s)^T(\mathbf{r}_m - \mathbf{r}_s)}{2w_s^2}\right], \tag{S.9}$$

where A_s was the signal amplitude, w_s was the signal width, and $\mathbf{r_s}$ was the center of signal.

The background object $\mathbf{f_b}$ was randomly sampled from a stochastic object model (SOM) which was generated by use of the progressively growing AmbientGAN (ProAmGAN) on a database of clinical brain MR images [3, 4].

The considered signal detection task was performed on a region of interest (ROI) of dimension of 64 $\times$ 64 pixels at the center of the reconstructed noisy image $\mathbf{f}_{recon}$. The two hypotheses for the task can be described as:

$$H_0 : \mathbf{f}_{recon} = |\mathbf{H}^{-1}(\mathbf{b} + \mathbf{n})|, \tag{S.10a}$$

$$H_1 : \mathbf{f}_{recon} = |\mathbf{H}^{-1}(\mathbf{b} + \mathbf{s} + \mathbf{n})|, \tag{S.10b}$$

where $\mathbf{b} = \mathbf{H}\mathbf{f_b}$, $\mathbf{s} = \mathbf{H}\mathbf{f_s}$, respectively.

One realization of the noise-free signal-present object $\mathbf{f}$, the inserted signal object $\mathbf{f_s}$, the reconstructed noisy image $\mathbf{f}_{recon}$, and the denoised estimate $\hat{\mathbf{f}}_{recon}$ are shown in Fig. S.3.

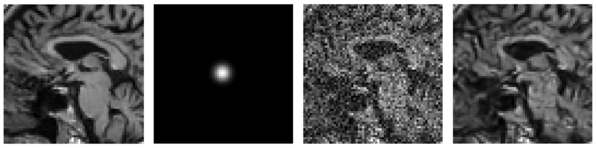

(a) (b) (c) (d)

Figure S.3: One realization of (a) the noise-free signal-present object $\mathbf{f}$; (b) the inserted signal object $\mathbf{f_s}$; (c) the reconstructed noisy images $\mathbf{f}_{recon}$; and (d) the denoised estimate $\hat{\mathbf{f}}_{recon}$ is displayed.

4.2 Numerical studies

The Gaussian signal defined in Eqn. (S.9) possessed an amplitude $A_s = 0.12$, width $w_s = \sqrt{2}$, and center location $\mathbf{r}_s = [32; 32]^T$. The dimensions of $\mathbf{s}$, $\mathbf{b}$, and $\mathbf{n}$ in Eqn. (S.10) were 64 $\times$ 64. The standard deviation of complex-valued Gaussian noise was

set to 8. Based on these settings, the training/validation/testing datasets were established and the CNN-based denoising networks of depth $D = \{3, 5, 7, 9, 11, 13\}$ were trained as described above in Sec. III.B.2 in the main article. The details of denoising networks training and observer computation are described in Sec. III.B.4 and Sec. III.C.4 in the main article, respectively.

4.3 Results

The impacts of depth of the trained denoising network on the NO performance are shown in Fig. S.4. It was observed that the NO performance on the denoised images yielded the same trends as were observed for the case where lumpy background model was employed. As such, these results are consistent with those shown in Fig. 6 in the main article.

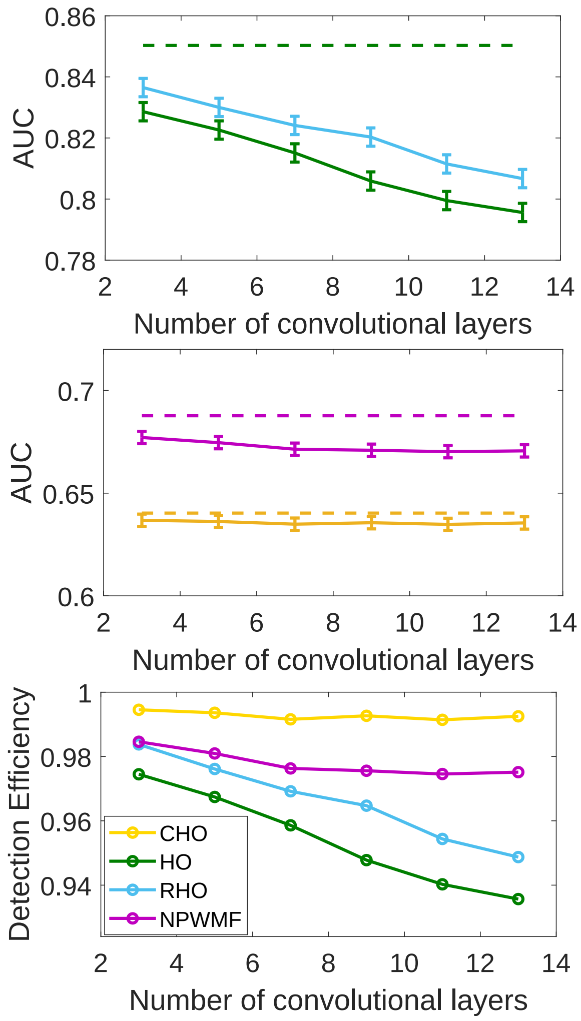

Figure S.4: The relationship between NO performance and the depth of the trained CNN-based non-linear denoising networks was quantified. The CNN-based denoising networks with the depths {3, 5, 7, 9, 11, 13} were employed in this study. The observer performance was quantified by AUC and signal detection efficiency. The three figures share the same legend. The dashed lines in the first two figures represent the performance of the NOs on the noisy images.

5 CNN-based observer test statistic approximation

Details regarding the implementation of the CNN-based observer test statistic are described below. The CNN-based observer network included a set of convolutional layers. Each convolutional layer comprised 64 filters with 5×5 spatial support followed by a Leaky ReLU activation function [5], a max-pooling layer [6] following the last convolutional layer was employed to sub-sample the feature maps, and a final fully connected (FC) layer used a sigmoid activation function to estimate the posterior probability probability $\Pr(H_1|\mathbf{g}, \Theta)$.

The train-validation-test scheme [7] was employed to train and evaluate the CNN-based observer. The initial training dataset included 150,000 noise-free signal-present images and 150,000 noise-free signal-absent images. To mitigate overfitting, a "semi-online learning" method [8,9] was employed in which measurement noise was generated on-the-fly and added to noiseless images drawn from the finite initial training dataset. The validation datatset included 200 signal-present images and 200 signal absent images and the corresponding noise-free target images. Finally, the test dataset comprised 10,000 signal-present images and 10,000 signal-absent noisy images. For training CNN-based observers on denoised images, the denoising operations were applied to the noisy images generated above to create denoised training data.

The CNN-based observer were trained by minimizing the cross entropy loss on mini-batches at each iteration. Each mini-batch contained 200 signal-absent images and 200 signal-present images randomly selected from the training data set. The Adam optimizer [10] with a learning rate of 0.00001 was employed for model training. For determining the optimal architecture of the CNN for approximating NOs, the training process starts from a CNN-architecture with 1 convolutional layer and gradually adds more layers. The optimal number of convolutional layers is determined when adding more layers does not significantly decrease the cross-entropy on the validation dataset. The cross-entropy was considered as significantly decreased if its decrements is at least 1.0% of that produced by the CNN-based observer with one less convolutional layer. The CNN-based observer having the minimum validation cross-entropy was selected as the optimal CNN-based observer in the explored architecture family. The training and implementation of CNN-based observers were performed using Tensorflow [11] on a single NVIDIA QUADRO RTX 8000 GPU.

References

[1] P.-C. Guo and Q. Ye, "On the regularization of convolutional kernel tensors in neural networks," *Linear and Multilinear Algebra*, pp. 1–13, 2020.

[2] X. Yi, "Asymptotic singular value distribution of linear convolutional layers," *arXiv preprint arXiv:2006.07117*, 2020.

[3] W. Zhou, S. Bhadra, F. J. Brooks, H. Li, and M. A. Anastasio, "Learning stochastic object models from medical imaging measurements using progressively-growing ambientgans." *arXiv preprint arXiv:2006.00033*, 2020.

[4] ——, "Progressively-growing ambientgans for learning stochastic object models from imaging measurements," in *Medical Imaging 2020: Image Perception, Observer Performance, and Technology Assessment*, vol. 11316. International Society for Optics and Photonics, 2020, p. 113160Q.

[5] J. T. Springenberg, A. Dosovitskiy, T. Brox, and M. Riedmiller, "Striving for simplicity: The all convolutional net," *arXiv preprint arXiv:1412.6806*, 2014.

[6] D. Scherer, A. Müller, and S. Behnke, "Evaluation of pooling operations in convolutional architectures for object recognition," in *International conference on artificial neural networks*. Springer, 2010, pp. 92–101.

[7] I. Goodfellow, Y. Bengio, A. Courville, and Y. Bengio, *Deep learning*. MIT press Cambridge, 2016, vol. 1, no. 2.

[8] W. Zhou, H. Li, and M. A. Anastasio, "Approximating the ideal observer and hotelling observer for binary signal detection tasks by use of supervised learning methods," *IEEE transactions on medical imaging*, vol. 38, no. 10, pp. 2456–2468, 2019.

[9] W. Zhou, H. Li, and M. A. Anastasio, "Approximating the ideal observer for joint signal detection and localization tasks by use of supervised learning methods," *IEEE Transactions on Medical Imaging*, vol. 39, no. 12, pp. 3992–4000, 2020.

[10] D. P. Kingma and J. Ba, "Adam: A method for stochastic optimization," *arXiv preprint arXiv:1412.6980*, 2014.

[11] M. Abadi, A. Agarwal, P. Barham, E. Brevdo, Z. Chen, C. Citro, G. S. Corrado, A. Davis, J. Dean, M. Devin *et al.*, "TensorFlow: Large-scale machine learning on heterogeneous systems," 2015, software available from tensorflow.org. [Online]. Available: https://www.tensorflow.org/
